# LoRa Power Model for Energy Optimization in IoT Applications

**DOI:** 10.3390/s26010301

**Published:** 2026-01-02

**Authors:** Juan Luis Soler-Fernández, Omar Romera, Angel Diéguez, Joan Daniel Prades, Oscar Alonso

**Affiliations:** 1 Department of Electronics and Biomedical Engineering, Universitat de Barcelona, 08028 Barcelona, Spain; oromera@ub.edu (O.R.); angel.dieguez@ub.edu (A.D.); 2 Institute of Semiconductor Technology (IHT), Technische Universität Braunschweig, D-38106 Braunschweig, Germany; daniel.prades@tu-braunschweig.de; 3 Laboratory for Emerging Nanometrology (LENA), Technische Universität Braunschweig, D-38106 Braunschweig, Germany

**Keywords:** LoRa, ultra-low power, energy harvesting, battery-less IoT nodes, power consumption model, remote sensing, ASIC controller, Python simulator

## Abstract

**Highlights:**

**What are the main findings?**

- We experimentally characterized all operating states (startup, transmission, reception, and sleep) of the Semtech SX1276 LoRa transceiver and built a parametric power model validated against measurements.
- The model captures the dependence on transmission power (RFO vs. PA_BOOST), sleep strategy (VCC ON/OFF) and packetization effects, and it remains configurable for the number of reception events.

**What are the implications of the main findings?**

- The model provides design guidelines for ultra-low power, harvested or battery-less IoT nodes, where minimizing the RF energy budget is critical.
- A distributable Python simulator based on the model allows other researchers to estimate system consumption and adapt the configuration to their own needs.

**Abstract:**

Energy efficiency is a key requirement for Internet of Things (IoT) nodes, particularly in applications powered by energy harvesting that operate without batteries. In this work, we present a parametric power model of a LoRa transceiver (Semtech SX1276) aimed at ultra-low power remote sensing scenarios. The transceiver was characterized in all relevant states (startup, transmission, reception, and sleep), and the results were used to build a state-based model that predicts average power consumption as a function of transmission power, sleep strategy, packetization, and input data rate. Experimental validation confirmed that the cubic fit for transmission peaks achieves a determination coefficient of 0.99, while reception is added as a constant consumption. The model was implemented in a Python simulator that provides mean, best-case, and worst-case estimates of system power consumption, and it was validated in an ASIC-based sensor node demonstration, with predictions within 10% of measured values. The framework highlights the trade-offs between energy efficiency and robustness (e.g., minimal SF and no CRC vs. higher spreading factors and error-control) and supports the design of custom controllers for ultra-low power IoT nodes as well as more energy-permissive applications.

## 1. Introduction

As the Internet of Things (IoT) continues to expand across sectors [1,2,3,4,5,6], the growing density of interconnected devices makes energy efficiency a key factor for ensuring the sustainability and longevity of these systems.

In smart cities, IoT systems collect and process data to address urban challenges such as traffic congestion, pollution, and resource management [7,8]. Networks of environmental sensors monitor air quality [9], noise levels [10], and weather conditions [11], providing valuable information to improve public health initiatives.

Home automation has been completely transformed by the incorporation of IoT technology into residential homes, which allows for intelligent control and connectivity of domestic spaces. This allows users to monitor and control their homes remotely using voice assistants or smartphones [12]. While these systems improve security with automated door locks and real-time surveillance, they also improve convenience with features like climate control and automated lighting [13]. In technical implementations, communication protocols like Wi-Fi for high-bandwidth applications or Zigbee for low-power mesh networks are frequently combined with inexpensive hardware (such as the Raspberry Pi and ESP32). Despite these advantages, interoperability, data privacy, and security issues still exist, requiring strong frameworks to safeguard private user information [14].

One key challenge of IoT solutions is the power consumption of connected devices. Many systems operate in remote or inaccessible locations [15,16,17], making it difficult or cost-expensive to replace and/or charge batteries frequently. Consequently, the development of low-power devices is imperative to extend the operational lifespan of these devices and reduce the environmental impact associated with frequent battery replacement. Moreover, some common applications such as wearables demand a small-sized system, leaving no space for large batteries.

Achieving low power in IoT devices involves a coordinated development of hard-ware, software, and communication protocols. Hardware optimization aims to minimize energy consumption during active and standby mode. Application-Specific Integrated Circuits (ASIC) allow for full control of the active circuits at every moment, giving results in the range of micro-Watts [18]. In addition, the hardware must include an efficient sensor working at the same micro-Watts level [19]. Likewise, software optimizations, including efficient algorithms, sleep modes, and duty cycling techniques, enable devices to save energy without compromising functionality or responsiveness.

Furthermore, an appropriate selection of the wireless communications protocol used in the system is crucial; it must allow the user to integrate the device into the ecosystem while maintaining a compromise between power consumption and range. Among the vast variety of existing protocols, we can highlight some of the most popular:

- Bluetooth Low Energy (BLE): is a protocol designed for short-range applications that require low latency and high data bandwidth. With its low energy consumption and compatibility with smartphones and other consumer devices, BLE finds its place in wearable devices, smart home appliances, and proximity-based applications such as indoor navigation.
- Long Range (LoRa): is a long-range and low-power protocol optimized for IoT applications requiring extended coverage and minimal power consumption. Operating in the sub-GHz frequency bands, LoRa enables communication over several kilometers in rural and urban environments [20,21], making it ideal for applications such as smart agriculture, environmental monitoring, and smart city infrastructure. LoRa’s robustness in challenging radio environments and its support for bidirectional communication further enhance its suitability for different IoT scenarios.
- Zigbee: is a low-power, low-data-rate protocol designed for short-range mesh networking applications. It offers reliable communication, low latency, and support for large-scale applications. Commonly used in smart home automation, industrial control systems, and building management, Zigbee facilitates interoperability among multiple devices and enables advanced features such as network routing and self-healing capabilities.
- Wi-Fi: While traditionally associated with high-speed internet access, Wi-Fi technologies have evolved to address the requirements of IoT applications. Wi-Fi HaLow (IEEE 802.11ah [22]) extends the range and lowers the power consumption of traditional Wi-Fi networks, making it suitable for IoT deployments in large-scale environments such as smart cities and industrial facilities. Wi-Fi’s ubiquity, interoperability, and high data throughput make it an attractive option for applications demanding real-time data streaming and high-bandwidth communication.
- NB-IoT (Narrowband IoT) and LTE-M (Long-Term Evolution for Machines): NB-IoT and LTE-M are cellular-based IoT technologies designed to provide low-power, wide-area connectivity for IoT devices. Leveraging existing cellular infrastructure, NB-IoT and LTE-M offer extensive coverage, reliable connectivity, and support for mobility, making them suitable for applications such as asset tracking, smart metering, and remote monitoring in areas where other wireless technologies may be impractical or unavailable.

Each wireless protocol presents advantages and disadvantages in terms of range, power consumption, data rate, scalability, and deployment cost. A careful evaluation of the requirements and constraints of a specific IoT application will decide the selection of the most appropriate protocol to optimize performance, energy efficiency, and overall system reliability.

To help system designers choose the most appropriate protocol in each situation, models of the wireless device are used to have a first approach of performance and energy consumption under different configurations and scenarios. Power models are used in the design phase, offering predictive results into how a device behaves under varying operational conditions. These models enable designers to estimate critical parameters, such as battery life, energy usage during transmission and reception, and the impact of duty cycles on power efficiency. By simulating real-world conditions, power models allow for the fine-tuning of hardware and software configurations before physical prototypes are built, significantly reducing development time and costs. They also help identify optimal trade-offs between communication range, data rate, and energy consumption, ensuring the final system meets the intended application’s requirements.

Furthermore, these models are crucial when developing custom ASICs, where achieving maximum energy efficiency is crucial. In such cases, power models guide the design decisions, ensuring the ASIC integrates seamlessly with the wireless device while adhering to the constraints of low power IoT applications.

Energy efficiency is the primary constraint for IoT nodes that harvest energy or operate battery-less [23]. Among LPWAN options, LoRa is attractive for its long range at modest power, yet end-node energy depends on radio states, transmit power path, packetization, and sleep strategy [24]. Existing studies often report time-on-air or average currents under specific settings, but few provide an experimentally parameterized, state-resolved model that designers can reuse across applications. Here we characterize all operating states of a LoRa transceiver (Semtech SX1276, Semtech Corporation, Camarillo, CA, USA), show that only the transmission peak current scales with output power, and fit this dependence with a compact polynomial. We integrate these data into a parametric model and release a Python (version 3.12.0 and run in Windows 10 version 10.0.19045.6456) simulator that predicts average power versus input data rate, transmit period, packet size, output path, and sleep strategy.

We validate the framework on an ASIC-based sensor node developed by our group, using it as a realistic platform to assess the model and discuss design rules that minimize energy while preserving link reliability. The ASIC implementation is not modeled itself; rather, it serves as a demonstration to validate the LoRa power model at the system level. The goal of the model is to provide ASIC designers with a predictive understanding of the wireless transceiver’s power behavior, so that system-level optimizations (e.g., duty-cycling, supply control, and data aggregation) can be defined early in the design process.

In this paper we present the PHY-level power model we used to develop a controller for the LoRa transceiver (Semtech SX1276) [25] device used in the development board, which we connected to an ultra-low power system-on-chip for IoT sensing nodes [18]. This model allows us to study the relation between the power consumption of the transceiver and its performance in terms of distance, packet size, etc. It is validated using performance data extracted from lab instruments for designing a custom controller and applying it to real-world applications in a sensor node. The model does not have into account possible regulations or legal limitations such as the 1% duty cycle limitation in the EU in the 433 MHz band.

The methodology here presented as well as the software implementation is not only limited to LoRa applications, but also extensible to other transceivers operating other wireless communication protocols as long as the current consumption of the device is characterized.

## 2. Materials and Methods

Our goal is to model the power consumption of a LoRa transceiver in the context of ultra-low-power remote sensing applications powered by harvested or scavenged energy, including battery-less scenarios. In such cases, the ability to transmit acquired data over RF links for as long as possible with a minimal energy budget is critical. To maximize efficiency, we configured the Semtech SX1276 device in its lowest-power operating mode, working in the 433 MHz band, disabling error-control features such as CRC and selecting the minimum spreading factor (SF). This configuration minimizes transmission energy, at the cost of discarding corrupted packets if channel conditions degrade. Based on these constraints, we designed a dedicated controller to operate the transceiver in the lowest-power configuration. At the same time, because other IoT applications may tolerate higher energy consumption in exchange for improved robustness, our model is kept general and parameterizable, allowing system designers to adapt it to their own requirements.

The experimental characterization used the minimal SF and with CRC disabled to minimize energy consumption. These settings define the scope of the experimental validation but do not restrict the model. Since the model computes energy as a combination of state durations and average currents, any change in SF or CRC modifies the duration of the transmission state. Therefore, users can adapt the model to other configurations by changing the data rate or the packet size parameters.

To quantify the transceiver consumption, we follow a state-based methodology, which sums up the contributions of its main operating states (e.g., startup, transmission, reception, and sleep) to determine the total energy needed by the transceiver. This method provides an intuitive framework for predicting average node power consumption because each state’s duration and current draw can be measured independently, then combined to yield an average power consumption.

For the characterization we used a SX1276MB1LAS_e311v02a development board powered by an Agilent B2912A source meter unit, which allows us to measure the current consumption of the board with a sampling rate of 10 μs.

**Figure 1:**
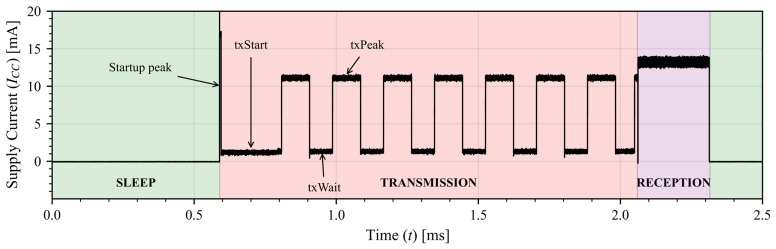
Semtech SX1276 current consumption. General view of an 8-packet transmission and reception in a 2 s window. Details of the different states are highlighted.

Figure 1 shows a record of the transceiver current draw for an 8-packet transmission and reception. We can identify multiple sections in the whole routine, coming from sleep mode in starts with a current peak “*startup peak*” and is followed by the “*txStart*” section, in which the transceiver prepares itself for a transmission. Then it starts sending the packets (“*txPeak*”) with a wait time between them (“*txWait*”), necessary to comply with the LoRa protocol. It ends with a fixed reception time window and goes back to sleep to start again in the future.

Current curves in Figure 1 shows the transceiver has four operation states with each one having a time duration and an average power consumption associated:

1. **Startup**: the power supply is enabled for the transceiver to boot up. $t_{\mathrm{startup}}$, $p_{\mathrm{startup}}$
2. **Transmission (tx)**:
  - **Start**: Wake up from sleep mode to transmission mode. $t_{tx_{\mathrm{start}}}$, $p_{tx_{\mathrm{start}}}$
  - **Peak**: Data transmission peak. $t_{tx_{\mathrm{peak}}}$, $p_{tx_{\mathrm{peak}}}$
  - **Wait**: time between packet transmission to ensure a proper reception. $t_{tx_{\mathrm{wait}}}$, $p_{tx_{\mathrm{wait}}}$
3. **Reception (rx)**: the device is set to work as a receiver. $t_{rx}$, $p_{rx}$
4. Sleep: time interval in which the radio is doing nothing. $t_{\mathrm{sleep}}$, $p_{\mathrm{sleep}}$

In these operating states, the transceiver’s behaviour across different scenarios can be characterized using five parameters:

1. **Radio transmission power**: low-power (0–14 dBm) RFO output mode but lower range or high-power PABOOST (2–20 dBm) output for long range.
2. **Sleep strategy**: power supply enabled during sleep periods (VCC ON) or power supply disabled during sleep periods (VCC OFF).
3. **Transmission period.**
4. **Data rate.**
5. **Number of packets per measurement interval.**

To evaluate each contribution to the final power consumption value we studied each variable independently from the others for the different states. We can differentiate two possible situations, using the low-power RFO output or the high-power PABOOST, and in both cases we can sweep the antenna power. The characterization covered the programmable power range of the transceiver in both output paths: RFO mode from 0 dBm to +14 dBm, and PABOOST mode from +2 dBm to +20 dBm.

**Figure 2:**
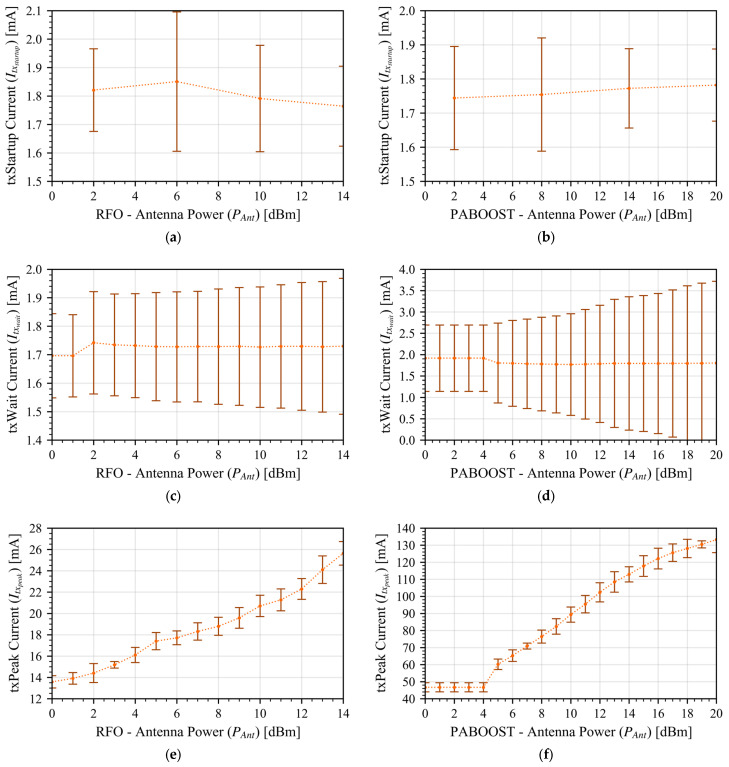
Transceiver average current consumption in all transceiver states for different antenna power values. Each point was measured 1000 times under same experimental conditions. The error bars correspond to one sigma deviation around the average. (**a**) txStartup, RFO. (**b**) Startup PABOOST. (**c**) txWait RFO. (**d**) txWait PABOOST. (**e**) txPeak RFO. (**f**) txPeak PABOOST.

Figure 2 shows the only section that is affected by the antenna transmission power sweep is the *txPeak* (Figure 2e,f); all other transmission states only depend on whether the RFO or the PABOOST is used. As expected, the low-power RFO output has a much lower power consumption.

To construct the model, we fitted the measured data using a cubic polynomial function, using only the odd data points for fitting while reserving the even points for later validation. After obtaining the average current consumption for each operating state, we developed a parametric model that calculates the average LoRa power over one complete operating cycle. A cycle includes (i) one startup event depending on the selected sleep strategy, (ii) transmission of the configured number of packets ($k_{\mathrm{packets}}$), (iii) optional reception intervals, and (iv) a sleep phase. This process yields the total energy budget per cycle, normalized by the cycle duration.

After each transmission sequence, the model includes an optional reception period that depends on the application requirements. This reception window is defined as a fixed duration long enough for the receiver node to send short reconfiguration data to the transmitter. Once this period ends, the system transitions to the sleep state. Not all applications require down-link communication; many energy-harvested IoT nodes operate in one-way transmission mode, such as environmental monitoring (e.g., air-quality sensors using LoRa in rural or urban deployments [26,27]) or structural-health monitoring systems where data is sent from sensors on infrastructure to a gateway without expecting frequent acknowledgements [28]. For those cases, the parameter can be set to zero in the model.

Each state power contribution is computed as

(1) $$P_{i}=I_{i}\cdot V_{CC}.$$

where $I_{i}$ represents the average current of a given state i and $V_{CC}$ is the supply voltage that is assumed to always be 3.3 V. All characterization measurements have been taken at this voltage.

During all measurements, although a receiver was connected listening to the transmitter, it can be considered transparent from the point of view of the transmitter power consumption because the receiver configuration will not affect the energy or timing of the transmission process.

The average power is then calculated for two possible sleep strategies, VCC ON and VCC OFF, which represents the power supply during the sleep time. ON means that the supply is kept active and the device is set to its own sleep, and OFF means that the power supply is completely shut off during the sleep intervals.

In the VCC OFF strategy, the transceiver supply line is disabled during the sleep time, meaning the device is fully powered down and its current consumption becomes negligible (≈0). When the supply is restored, the radio has a startup peak before the next transmission, increasing the startup time and slightly raising the energy per cycle.

In contrast, the VCC ON strategy keeps the transceiver’s supply active while setting the device into its internal sleep mode. This configuration maintains a small residual current consumption but avoids the startup peak.

These two approaches define a design trade-off: VCC OFF minimizes average energy usage over long sleep intervals, whereas VCC ON is advantageous when frequent wakeups are required.

VCC ON:

(2) $$P_{VCC\;ON}=\frac{p_{startup}t_{startup}+p_{tx}t_{tx}+p_{rx}t_{rx}+p_{sleep}t_{sleep}}{t_{startup}+t_{tx}+t_{rx}+t_{sleep}}$$

where the transmission contribution $p_{tx}$ is calculated as the average power over all transmission subsections during a transmission sequence. The transmission state consists of three parts: a single *start* phase followed by $k_{packets}$ repetitions of the packet transmission *peak* and inter-packet *wait* period:

(3) $$p_{tx}=\frac{p_{tx_{start}}t_{tx_{start}}+k_{packets}\cdot \left(p_{tx_{peak}}t_{tx_{peak}}+p_{tx_{wait}}t_{tx_{wait}}\right)}{t_{tx_{start}}+k_{packets}\cdot \left(t_{tx_{peak}}+t_{tx_{wait}}\right)}$$

VCC OFF:

(4) $$P_{VCC\;OFF}=\frac{p_{startup}t_{startup}+p_{tx}t_{tx}+p_{rx}t_{rx}}{t_{startup}+t_{tx}+t_{rx}}$$

In this scenario, the module is shut off in the sleep time, effectively reducing the power in this state ($p_{sleep}$) to zero.

The model is developed in Python to implement the aforementioned equations and states characterization. The user inputs the parameters presented in Table 1, the script takes the inputs and calculates the number of packets ($k_{packets}$) from the values of DRin, Bytes and T; e.g., a system capturing a 16 bit measurement every second (DRin=16) for 15 min would send a total of 1800 bytes, with a number of bytes per packet of 255, $k_{packet}$ would result in 8 packets.

The Python simulator calculates the mean, best-case, and worst-case power consumption by considering the observed variation in the measured current values for each operating state and then plots the corresponding curves.

## 3. Results and Validation

**Figure 3:**
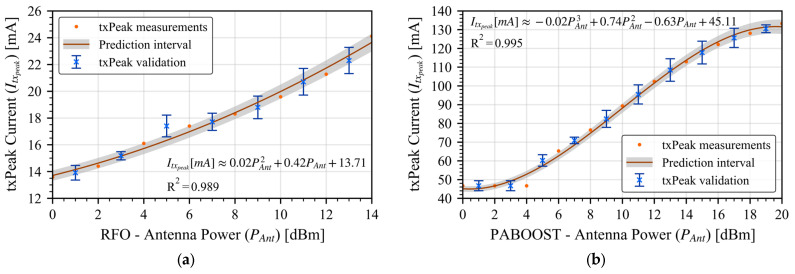
*txPeak* characterization and polynomial fitting extraction validation. (**a**) RFO output. (**b**) PABOOST output.

As shown before, *txPeak* is the only state with a strong dependence on the transmission power, for this reason we characterized it and validated it independently. All points have been captured 1000 times to have a statistical variation. We fitted a cubic polynomial function to the even measurements and validated the interpolation with the odd values. Results are presented in Figure 3 for both output cases, RFO and PA_BOOST; the fitting predictions align well with the measured data (all points lie within the standard deviation error bars) with an R^2^ of 0.99, thus confirming the model’s ability to capture the *txPeak* current for any power setting between the measured boundaries.

In addition to transmission, we also characterized the reception (PHY-only) state which revealed that the transmission antenna power has no effects in the power consumption of the reception side and it keeps constant current consumption the whole time the radio is set to receiver. For this reason, we have a constant current for the RX time of 13.2 mA (average across the reception window) that lasts for 350 ms after each transmission (as shown in Figure 1). The time window that the reception is active can be modified in the model to suit other applications requirements, in our case this time was the minimum necessary to receive information that the system can process.

With the model written we started studying the transceiver behavior with the goal of developing a custom controller for an ASIC [29] with all the optimal values to reduce power consumption while maintaining functionality.

**Figure 4:**
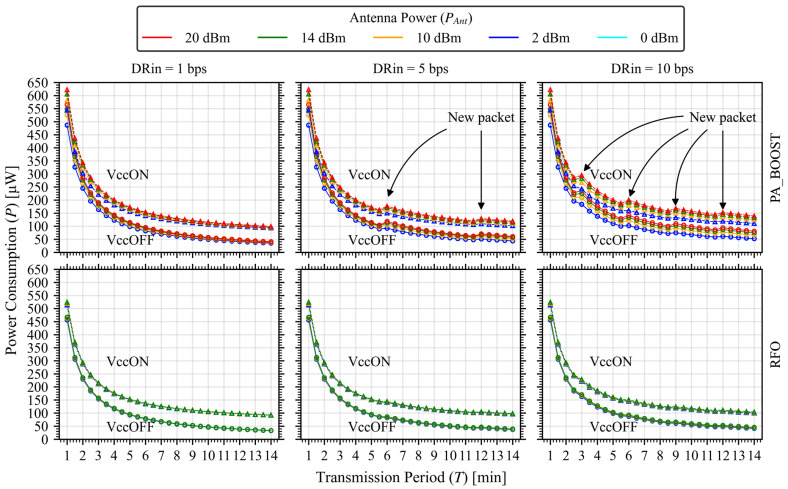
Multiple configurations example results of the power model. Each graph is evaluated for multiple antenna power and swept over the transmission period. Different sleep strategies are also embedded in the curves (triangles for VCC ON and circles for VCC OFF) with a supply voltage of 3.3 V. Upper curves: PA_BOOST output. Lower curves: RFO output.

In Figure 4 we present an example of usage, where we study the effects of varying the transmission period T under different transmission powers, output powers (RFO vs. PA_BOOST), data rates, and sleep strategies. For a fixed input data rate (*DR_*in*_*), the payload accumulated over T is packetized into k packets, with

(5) $$k_{packets}=ceiling\left(\frac{DR_{in}\cdot T}{255}\right).$$

The combination of values that generate a new packet in the transmission stream are marked with an arrow and, with a closer look, a step up in power consumption is generated, giving us higher power consumption for less data.

In Figure 4, the “antenna power” values in dBm correspond to the RF output power generated by the transceiver’s internal amplifier. This value represents the signal power applied to the antenna rather than the effective radiated power, and it is set through the device’s two available output paths: the RFO output, and the PA_BOOST output (high-power mode). Since the radiation pattern and antenna gain depend on the external antenna design and do not affect the electrical consumption of the transceiver, they were not included in the power model. These parameters influence only the link efficiency and received signal level, not the transmitter’s internal energy behavior.

With the characterization of all relevant states (startup, transmission, reception, and sleep), we integrated the results into a parametric model of the SX1276 transceiver. To make this model easily accessible, we developed a distributable LoRa Power Consumption Simulator to share the model of the LoRa module with the community [30]. It enables the user to have an approximate value of the overall system consumption in IoT environments.

The simulator uses a command-controlled application runnable in a Python environment. To use it, you only need the Python script “LoRa_power_model.py” and a custom dataset which includes all experimental parameters ready to work. As inputs, the simulator needs the use conditions and free variables described in the model. It returns the predicted mean power consumption as well as the best and worst cases with their related uncertainties.

**Figure 5:**
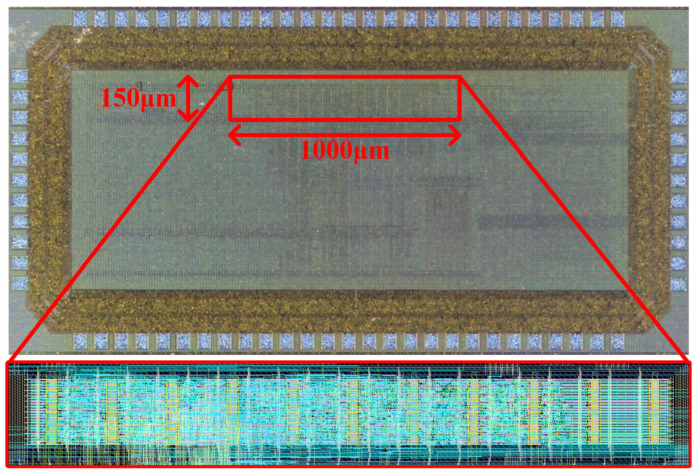
(**Top**) microphotograph of the whole ASIC that includes the custom Semtech SX1276 LoRa controller. (**Bottom**) layout of the controller section with a total area of 1000 μm × 150 μm.

We used all this data to design a custom controller incorporated into an ASIC (Figure 5) [29] intended for ultra-low power sensor monitoring. The system acquires sensor measurements every second (i.e., 1 Hz frequency) and transmits aggregated data packets every 15 min to a receiver node. From the transceiver perspective, it is translated to sending 900 measurements per transmission cycle, equivalent to 1800 bytes payload (2 bytes per measurement), at 15-min intervals.

To minimize power consumption, the controller implements three key optimizations extracted from the simulator:

1. Transmit Power Reduction: Antenna output power is configured at the minimum viable level within the characterized range (0 dBm) to maintain reliable communication,
2. Timing Optimization: Transmission dead time is minimized, and a 0.3 s reception window is enforced after each transmission for system reconfiguration,
3. Sleep Strategy: The transceiver’s power supply (VCC) is completely switched off between transmission cycles. For extended 15 min sleep intervals, this approach, despite forcing periodic startup current peaks, proves more efficient than maintaining VCC ON, as it eliminates standby leakage current that would otherwise dominate the power budget (Figure 4).

**Figure 6:**
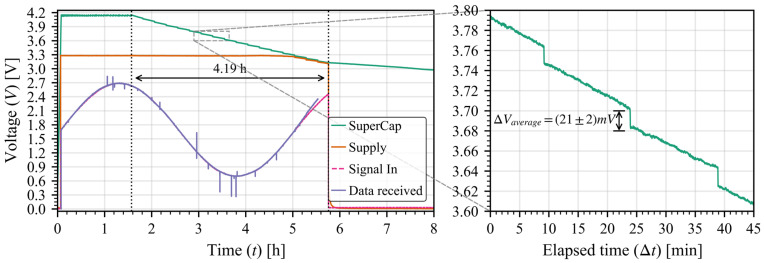
Super-capacitor discharge (green) as a function of time with a transmission every 15 min of 1800 bytes (packets of 255 bytes) for an antenna power of 0 dBm. The gray dashed line delimits a zoom in of the whole curve to capture the voltage steps of each transmission.

The ASIC was set up to measure a controlled voltage signal over time while sending all the data recollected every 15 min. It was supplied with a 470 mF super-capacitor at a maximum voltage of 4.2 V with a regulator to generate the 3.3 V supply, the capacitor voltage was tracked to see the overall power consumption of the system. Results are presented in Figure 6 where we can see the effects of each transmission, resulting in a (21 ± 2) mV voltage drop (averaged over the full discharge) from the super-capacitor. With the voltage drop we can measure the energy usage per transmission ($E_{TX}$) and the weighted power per transmission ($P_{TX}$), where Δt corresponds to the time interval between transmissions which corresponds to the parameter *Transmission period* (T) in the model:

(6) $$E_{TX}=\frac{1}{2}C\left(V_{i}^{2}-V_{f}^{2}\right)$$

(7) $$P_{TX}=\frac{E_{TX}}{\Delta t}$$

For a 470 mF capacitor, this results in a total measured weighted power consumption of (40 ± 3) μW.

**Figure 7:**
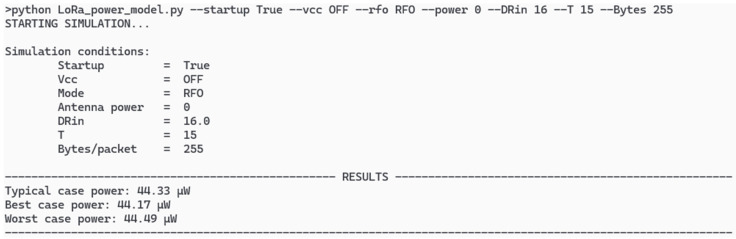
Python command-line interface simulator for the conditions of the deployment test of the ASIC with an antenna power of 0 dBm.

We previously entered the test conditions into the model. Figure 7 presents the Python simulator showing the command-line interface in which the user sets the variables needed to run the model and it returns the predicted power consumption for those conditions in which resulted to 44.33 μW typical power. The predicted value differs with the measurements in less than 1 μW considering the errors involved in both cases. The slight variations with the real-world measurements in the system come from the incompleteness of the eighth packet, which is counted as a full 255-bytes packet, although it only sends the last 15 bytes.

The predicted results show a narrow difference between the best and worst cases due to the static currents in each state in which there are almost no transient currents for most of the time.

**Figure 8:**
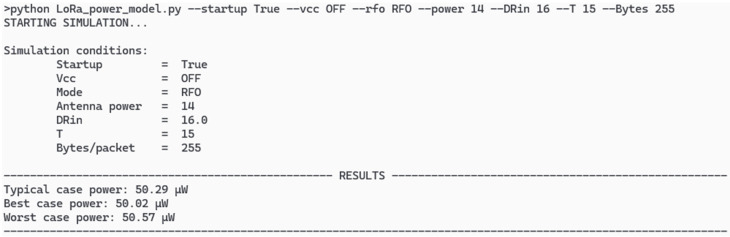
Python command-line interface simulator for the conditions of the deployment test of the ASIC with an antenna power of 14 dBm.

**Figure 9:**
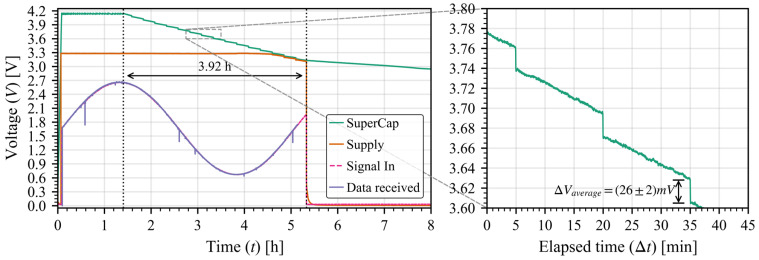
Super-capacitor discharge (green) as a function of time with a transmission every 15 min of 1800 bytes (packets of 255 bytes) for an antenna power of 14 dBm. The gray dashed line delimits a zoom in of the whole curve to capture the voltage steps of each transmission.

In order to widen the verification of the model, we tried the same test but changed the antenna output power to 14 dBm in RFO mode; the model results are presented in Figure 8 and the real-world measurements are shown in Figure 9. The (26 ± 2) mV drop corresponds with a weighted power consumption of (48 ± 2) μW; as in the previous validation case, both results are well within useful range for a prediction scenario, and the slight variation again comes from the incompleteness of the last packet Also, the “alive” time is shorter due to the higher power consumption of the communications (as the rest of the circuits are the same).

## 4. Discussion

The proposed state-based model replicates the transceiver’s behavior by decomposing a transmission cycle into startup, transmission (txStart/txPeak/txWait), optional reception, and sleep. The only state that varies strongly with antenna power is txPeak, as experimentally characterized for both RFO and PA_BOOST paths; a cubic fit captures this dependence with $R^{2}\approx 0.99$ and the validation points fall within the measured standard-deviation bars, supporting the use of this fit inside the model’s valid power ranges. The remaining states depend primarily on the selected output path (RFO vs. PA_BOOST) and the sleep strategy (VCC ON/OFF), which the model treats explicitly. All results assume a regulated VCC=3.3 V.

Building on this basis, sweeping the transmission period T reveals two competing effects. Increasing T amortizes startup, lowering average power. However, as data accumulates between transmissions, the packet count $k_{packets}$ increases in steps whenever the aggregated payload exceeds the packet size, introducing discrete rises in energy per cycle. In our curves, this yields the familiar decrease-then-rise pattern in average power; the minima occur just before a $k_{packets}$ increment, especially when VCC is OFF, because startup is then fully amortized across a longer sleep. Conversely, when VCC is ON the optimum shifts depending on the sleep current.

These effects manifest differently depending on the output stage. With RFO, per-packet energy is low, so the staircase caused by $k_{packets}$ increments is mild and the curves appear smooth, trending toward a shallow minimum as T increases. With PA_BOOST, per-packet energy is higher, so each $k_{packets}$ increment has a larger impact and the non-monotonic behavior becomes pronounced: at higher data acquisition rates ($DR_{in}\approx 5-10\;bps$ in our examples), waiting longer can increase average power if the longer period triggers one extra packet. This explains why the PA_BOOST curves in Figure 4 exhibit step-ups while the RFO curves look nearly monotonic.

From these observations, several design implications follow. First, RFO should be preferred whenever the link budget allows, since the energy penalty of PA_BOOST is visible at the cycle level. Second, for a given DRin and packet size, it is beneficial to select a period T just below the next packetization threshold to avoid stepping from k to k+1. Third, VCC-OFF is advantageous when T is long enough to amortize startup; otherwise, VCC-ON can be competitive due to avoided re-initialization.

While reception has a fixed and relatively small energy contribution in our configuration (one packet per cycle), in other deployments where multiple downlink messages are expected, its effect can accumulate. The model allows this parameter to be scaled, so designers can explore the trade-off between robustness (more receptions) and energy budget.

Finally, to validate the model at the system level, we compared its predictions with field data. Deploying the controller ASIC with an SX1276 node that acquires one 2-byte reading per second and transmits 1800 bytes every 15 min (≈8 packets at 255 bytes each), we observed ~35 mJ per transmission event (≈21 mV drop on 470 mF), which corresponds to ~41 µW weighted over a 15-min cycle. Under those same conditions, the Python simulator predicted 44.3 µW. Increasing the antenna power to 14 dBm produced a larger voltage drop of (26 ± 2) mV, equivalent to (48 ± 2) µW. Under these same conditions, the Python simulator predicted ≈49 µW. The higher estimate is small and consistent with measurement variability and unmodeled system elements (e.g., DC/DC conversion behavior over light loads, temperature drift, or exact packetization overhead of the last remaining bytes that are counted as a full packet), supporting the use of the model for early-stage design and scheduling.

The comparison with the ASIC prototype closes the validation loop between the modeled transceiver behavior and the real integrated system. Although the ASIC itself is not part of the power model, it provides a representative implementation context to verify that the predicted transceiver energy aligns with full-system measurements. This validates the model as a design aid for ASIC and embedded system engineers who must anticipate wireless energy costs before hardware integration.

These observations are especially relevant for battery-less sensor nodes, where the entire RF budget must be supplied by intermittent sources. Under such constraints, our minimal LoRa configuration (no CRC, lowest SF) favors energy efficiency over link robustness, accepting the loss of some packets to extend node lifetime. While this trade-off is appropriate for our use case, the model remains flexible; users with more generous energy budgets can increase the SF, which effectively raises the number of bits per message and thus the required DRin, and the state-based framework will still provide reliable power estimates.

To emphasize, while the measurements reported here were taken with CRC disabled and the minimum SF to represent the most energy-efficient case, the model remains valid for other configurations. Adjusting the Data Rate or the packet size parameter, to increase the transmission time, allows the same framework to estimate power consumption for CRC-enabled or higher-SF modes.

In the context of energy-harvesting operation, the supercapacitor is used as a short-term energy buffer rather than as a long-term storage element. Its function is to temporarily accumulate the harvested energy and supply the instantaneous power required for high-current events such as LoRa transmissions, even when the harvester output fluctuates. Although supercapacitors are less common than rechargeable batteries in IoT nodes, studies have employed them in energy-harvested wireless systems where fast charge/discharge capability and high endurance are critical [31]. Compared with batteries, supercapacitors exhibit lower energy density and higher self-discharge, but they provide fast response, long cycle life, and maintenance-free operation, while using materials with lower environmental impact [32]. In addition, their nearly ideal capacitive behavior simplifies the energy consumption measurements, allowing more accurate and repeatable power profiling than with electrochemical batteries, whose internal voltage and efficiency vary with state of charge. These characteristics make supercapacitors a suitable choice for autonomous and hard-to-access sensor nodes where energy is harvested intermittently and burst-type power delivery is required.

Naturally, there are limitations. The presented model focuses on LoRa PHY device states and excludes protocol-level events such as join procedures, adaptive data rate (ADR) adjustments, or downlink windows, which can add further reception phases. Measurements were conducted under regulated lab conditions at 3.3 V; factors such as temperature variation, supply fluctuations, or DC/DC efficiency at ultra-light loads may shift the observed optima. Characterizing the device under real environment conditions, including harvested power profiles, would further refine the model’s accuracy. Looking ahead, future work may extend the framework to newer LoRa transceiver families (e.g., SX126x) and integrate these environmental and protocol-level factors, broadening the applicability of the model to real-world IoT deployments.

## 5. Conclusions

We presented a state-based power model for a LoRa transceiver (SX1276), experimentally characterized in all operating states and validated with measurements. The model, implemented in a distributable Python simulator, enables accurate estimation of average consumption under different configurations and guides the design of ultra-low power or battery-less IoT nodes. Validation in an ASIC-based sensor node confirmed predictions within ~10% of measured values, supporting its use for early-stage design and energy budgeting. Future work (along with the community) may extend the approach to newer LoRa families and to protocol-level events such as LoRaWAN downlink windows. Also, temperature dependance would be an interesting addition, especially for outdoor applications where the day is much warmer than the night.

## Figures and Tables

**Table 1:** Python simulator parameters list.

Symbol	Parameter	Description
VCC	Sleep strategy	Can be ‘ON’ or ‘OFF’. It selects whether the power supply is turned on or off during sleep periods, respectively.
–	Startup	If true, startup current peak is included. If false, it is not.
RFO	RFO	If ‘RFO’, the transmitter uses the lower power configuration (from 0 dBm to 14 dBm. If ‘PABOOST’, the transmitter uses the power amplifier configuration (2 dBm to 20 dBm).
$P_{\mathrm{Ant}}$	Antenna power	Antenna power output. It must be inside the range of the RFO parameter configuration.
T	Transmission period	Period between two consecutive transmissions in minutes.
$DR_{\mathrm{in}}$	Data rate input	Data bits captured per second from the system.
–	Bytes	Number of bytes per packet.

## Data Availability

All data used to construct the power consumption model and Python simulator can be found in the public GitHub repository [30].

## Abbreviations

The following abbreviations are used in this manuscript:

ADR	Adaptive Data Rate
ASIC	Application Specific Integrated Circuit
BLE	Bluetooth Low Energy
IoT	Internet of Things
LoRa	Long Range
Rx	Reception
SF	Spreading Factor
Tx	Transmission
